# Self-censorship: should scientific journals decline to publish self-experimentation?

**DOI:** 10.1136/jme-2025-110730

**Published:** 2025-09-10

**Authors:** Jonathan Pugh, Dominic Wilkinson, Julian Savulescu

**Affiliations:** 1 Uehiro Oxford Institute, University of Oxford, Oxford, UK; 2 Centre for Biomedical Ethics, Yong Loo Lin School of Medicine, National University of Singapore, Singapore; 3 Newborn Care, John Radcliffe Hospital, Oxford, UK; 4 Murdoch Children’s Research Institute, Melbourne, Victoria, Australia; 5 Department of Paediatrics, School of Clinical Sciences, Faculty of Medicine Nursing and Health Sciences, Monash University, Melbourne, Victoria, Australia

## Abstract

A virologist recently made headlines after successfully using an experimental form of oncolytic virotherapy to treat her own recurrent breast cancer. This case has come at a time when regulators are increasingly having to grapple with the proliferation of self-experimentation outside of accredited research institutions. There is, therefore, a pressing need to outline the key ethical dimensions of self-experimentation and to develop ethical guidance for journals that may be faced with decisions about whether to publish research involving self-experimentation. In this paper, we aim to provide such guidance. We argue that while self-experimentation is not always ethically problematic, neither is there an in-principle moral reason for exempting it from ethical evaluation. After summarising the details of the recent case report of self-experimentation and briefly placing it in historical context, we suggest that it is possible to navigate the ethical issues raised in cases of self-experimentation by returning to fundamental values in research ethics, focusing on the implications of self-experimentation for respect for autonomy, reasonable risk, and preventing harm to others. We apply these principles to the case report and explain why the publication of this report can be morally justified. We ultimately advocate for a case-by-case assessment of studies involving self-experimentation submitted for publication by ethical review boards and journal editors, and we propose a decision-making algorithm to help guide such decisions.

The recently publicised case of Beata Halassy prompted widespread discussion of the ethics of self-experimentation and whether there are any ethical concerns with publishing scientific findings from such experiments.^1 2^ Indeed, one notable feature is that Halassy reported having encountered difficulties in seeking to publish the findings of the experiment, due to concerns raised about the ethics of self-experimentation.^1^

Halassy is not the first notable self-experimenter in medicine, and she is unlikely to be the last. Indeed, the case has come at a time when regulators are increasingly having to grapple with the proliferation of biological self-experimentation outside of accredited research institutions, among so-called ‘biohacker’ communities.^3^ This is due in part to the increasing consumer availability of biotechnological tools (such as the gene-editing tool CRISPR-Cas9) and the move towards open-source science.^4^

Accordingly, there is a pressing need to outline the key ethical dimensions of self-experimentation, and to develop ethical guidance for journals that may be faced with decisions about whether to publish research involving self-experimentation.

Our aim in this paper is to provide such guidance. In doing so, we shall explain that although some cases of self-experimentation may raise legitimate ethical concerns, self-experimentation is not always ethically problematic. However, neither is there an in-principle moral reason for exempting self-experimentation from ethical evaluation. Accordingly, decisions about whether to publish research involving self-experimentation should be made on a case-by-case basis. We outline key considerations that should factor into such decisions and explain how they speak in favour of the publication of the report detailing Halassy’s case. We begin by providing an overview of key information detailed in the case report of her experiment, previously referenced in news reports on Halassy’s story.^1^

## A summary of the case report

Halassy’s self-experimentation involved the experimental use of oncolytic virotherapy (OVT) in the treatment of a recurrent breast cancer.^5^ OVT is an emerging approach to cancer therapy, grounded in the insight that certain viruses have an innate ability to infect and preferentially destroy tumour cells.^6^ Although OVT is a promising avenue of research in cancer treatment and the subject of ongoing clinical trials (including in breast cancer), the authors of the case report note that the results of clinical studies of OVT are ‘often significantly below what is expected and fail to repeat…preclinical success’.^5^

The published case report of the experiment includes a case history, which details that ‘the patient was a 50-year-old woman with a history of local recurrence of triple-negative breast cancer’ following an initial diagnosis of the tumour in 2016. It explains that the patient (Halassy) was initially treated with mastectomy followed by adjuvant chemotherapy and that a small local recurrence was also surgically removed; however, a small seroma remained, which would require further monitoring. By 2020, this structure had progressed to a solid tumour, and further testing revealed that it had invaded into the pectoral muscle.^5^

Although Halassy has stressed in media reports that she is not a specialist in OVT,^1^ the case report clarified that she is a professional virologist ‘with expertise in growing and characterising human and animal viruses’.^5^ Aware that existing therapies have limited efficacy for her type of cancer, the case report details that she informed her oncologist that she would treat this tumour with ‘intratumoural injections of research-grade virus preparations, which first included an Edmonston-Zagreb measles vaccine strain and then a vesicular stomatitis virus Indiana strain (VSV), both prepared in her own laboratory’.^5^ Her oncologist agreed to monitor her progress, and OVT was initiated after necessary baseline tests had been performed. The report details that she did not experience any serious side effects during the course of treatment, but the tumour significantly reduced in size after only 2 months of therapy.^5^ Following treatment, the tumour was no longer infiltrating the muscle below or skin above, and it was successfully excised. Having also completed appropriate adjuvant therapy, Halassy had been in remission for 45 months at the time of the report’s publication. The authors conclude by encouraging formal clinical trials assessing OVT as neoadjuvant therapy in early cancer, while acknowledging the isolated and unconventional nature of the case report.^5^

The case report also provides a statement from the Institutional Review Board (IRB), which stated that the case report does not require ethics committee review as it is a ‘case of self-experimentation’.^5^ However, it clarifies that the patient was fully informed of her illness and available therapies, that she wanted to pursue an ‘innovative approach in a scientifically sound way’ and that her progress was being monitored by her oncologist, who would intervene ‘with conventional therapy if there were untoward effects or if the tumour progressed’. Finally, the report includes a statement clarifying that ‘informed consent was obtained’ from the patient involved.^5^

## The case in context

Halassy’s case is the latest in a long history of self-experimentation, with documented examples predating 1800.^7^ Space here only allows for a brief sketch of some particularly notable cases. These include a number of success stories, with self-experimentation involving work of such value that it led to the experimenter winning a Nobel Prize.

Among these is Niels Finsen, who discovered key principles of phototherapy, partly via his own attempts at self-treatment.^8^ Werner Forssmann shared the 1956 Nobel Prize in medicine or physiology for discoveries concerning heart catheterisation, having successfully performed the procedure on himself in 1929.^7^ More recently, in 2005, Barry Marshall also won a Nobel Prize after he demonstrated the role of *Helicobacter pylori* in the development of peptic ulcers by ingesting the bacterium. This, in turn, led to him developing gastritis, which he treated with antibiotics. It is estimated that this work has saved millions of lives.^9^ However, the history of self-experimentation also includes a number of cautionary tales. In his overview of the history of self-experimentation, Weisse highlights eight scientists who died as a direct result of their self-experiments (often in cases involving infectious disease), including Jesse Lazear, who was involved in the 1900 Reed Commission experiments identifying mosquitoes as the vector of yellow fever. Weisse also notes that others have been affected by various kinds of negative sequelae as a result of their self-experimentation.^7^

Self-experimentation has also made more recent headlines. During the COVID-19 pandemic, a collective of citizen scientists named ‘Rapid Deployment Vaccine Collaborative’ (RaDVac) announced that they aimed to rapidly develop, produce and self-administer an intranasally delivered COVID-19 vaccine, prompting academic discussion of the regulation of self-experimentation.^10^ Furthermore, in 2017, a biohacker named Jo Zayner injected herself with a DIY CRISPR-Cas9 gene therapy, aiming to enhance muscle growth, leading the US Food and Drug Administration to issue warnings about the dangers of such self-experimentation.^11^

## The performance question and the publication question

The ethics of self-experimentation can raise novel and complex ethical issues. Weisse reports that after sending letters of enquiry, he discovered that some IRBs might decline to approve any form of self-experimentation.^7^ Others may regard such studies as falling outside their remit for reasons we shall explore below. Part of our aim in this paper is to suggest that both such responses are mistaken.

In order to navigate these issues, it is useful to distinguish two distinct questions:[^i^]^12^

1. *The performance question*. Is it ethical to perform an experiment on oneself?
2. *The publication question*. Is it ethical to publish the findings of such an experiment as a form of scientific research?

The reason that it is important to distinguish these questions is that activities that fall within the domain of scientific research are bound by ethical norms that are more restrictive than those that govern our actions more broadly. In liberal societies, it might be thought that if the performance of self-experimentation only poses a risk of harm to the experimenter herself, then third parties should not have the authority to prevent the individual from deciding to expose themselves to the risks of self-experimentation. This idea is enshrined in Mill’s ‘Harm Principle’, which states that ‘the only purpose for which power can be rightfully exercised over any member of a civilised community, against his will, is to prevent harm to others’.^13^ However, even if we assume that the performance question should be answered by appeal to liberal ideals of non-interference, it is far from clear that such ideals should also be invoked with respect to the publication question. That is because the dissemination of information as ‘scientific research’ can impact others (in both beneficial and harmful ways).

In order to develop this point further, it will be useful to return to the fundamental values that govern scientific research, and that ethics committees are primarily in place to safeguard. Prior to doing so, though, it is first important to address a perhaps more fundamental issue: namely, whether cases of self-experimentation do in fact amount to scientific research.

## Is self-experimentation research?

Settling the vexed question of how to appropriately demarcate the boundaries of what constitutes research is beyond the scope of this paper. For our purposes, we shall rely on Kolstoe *et al*’s recent analysis.^14^ Kolstoe *et al* highlight that research can be distinguished from other activities that typically do not require IRB review (such as health surveillance and clinical audit) by considering a number of factors, three of which we shall particularly highlight here; namely, (1) the purpose of the activity, (2) the interventions it involves and (3) the data that is collected. Briefly, research will typically involve the planned use of a documented methodology that will allow results to be generalised and transferred to a larger population. It may (but need not) involve evaluating interventions, particularly new ones, and it will usually (though not always) involve collecting and analysing data that are additional to those obtained for routine care. On this approach, some examples of self-experimentation (including some mentioned above) might plausibly qualify as ‘research’.

However, other examples may not. Indeed, many of the self-experiments listed above were plausibly pursued with a therapeutic, rather than scientific, purpose; this is true, even if the success of the intervention led later to the pursuit of publication. Other self-experiments (eg, biohackers) may have the intention to enhance normal function, rather than to treat an illness or disease, but they equally are not necessarily aiming to produce generalisable knowledge.

This difference matters because it is widely accepted that ethical scientific research must be preceded by IRB review. Such review addresses the distinctive ethical considerations raised by both (1) the activities involved in the research and (2) the publication of the results generated. When a self-experiment straight-forwardly qualifies as ‘research’, it is difficult to see why it should not be subject to the kind of ethical oversight that is understood to be appropriate for that kind of activity. However, as Kolstoe *et al* point out, since IRB review can be time-consuming and costly, there are good reasons not to classify activities as research if this is not necessary (i.e, if they are appropriately covered by other ethical principles).^14^

Our suggestion is that the *mere performance* of a self-experiment need not always be preceded by IRB review, since such activities can appropriately be covered by other ethical principles. In some cases, this might be because the self-experiment was pursued as a therapeutic intervention within an institutional setting, where the performance of the activity is covered by the governance, guidelines and ethical principles that accompany usual medical care. Outside the walls of healthcare institutions, the performance of a self-experiment that does not clearly qualify as research and that does not occur as part of medical care is, we suggest, most appropriately governed by liberal norms of non-interference.

Yet, we believe that it would be a mistake to preclude the publication of the experiment solely on the basis that the performance of the activities involved did not receive prior IRB approval.[^ii^]^15 16^ Instead, what matters when we consider the publication question with respect to these self-experiments is whether those activities were compatible with the norms and fundamental values of research. It is to these fundamental values we now turn.

## Back to fundamental values in research ethics

### Respect for autonomy

The principle of respect for autonomy is a cornerstone of both research and clinical ethics, and the informed consent process is a crucial protection in both contexts. When considering research involving individuals with decision-making capacity that exposes them to a non-minimal degree of risk, it is crucial that participants have provided sufficiently informed and voluntary consent to participation in a research trial. Considerations of autonomy might also plausibly be central to the performance question outside of medical contexts for those who believe that liberal societies ought only to respect an individual’s decision to engage in risky behaviour if their choice was autonomous. However, we shall set that question aside here.

One of the perennial debates in research ethics concerns how much information researchers must disclose to participants, and which standards of disclosure should be invoked in the context of clinical research.^17–19^ One complicating factor in self-experimentation is that one individual is playing the ‘dual role’ of both researcher and participant; they may thus share some of the vulnerabilities associated with typical research participants, but they also share the responsibilities of the researcher. In view of this dual role, the pertinent question about respect for autonomy in self-experimentation is not so much whether the researcher has discharged their duty to disclose relevant information; rather, the pertinent question is whether the individual has sufficient understanding to make an autonomous decision to undergo the experimental intervention.

In Hallasy’s case, the paper details that she gave informed consent and that she understood material information about the decision to undergo the experimental therapy. So, there are plausible grounds for believing that her decision to undergo the self-experiment was made on the basis of a good understanding of her choice. Similarly, in many cases of historical self-experimentation, the individuals involved have been highly knowledgeable in their field, and it seems plausible that these individuals will also have had a good understanding of what they were doing; Barry Marshall, for instance, had a much more accurate understanding of the likely consequences of ingesting a *H. pylori* culture than contemporaneous medical professionals who doubted its role in the development of peptic ulcers. Accordingly, in some cases, it appears challenging to mount a convincing argument that self-experimenters lacked sufficient understanding to make an autonomous choice to undergo their experimental intervention.

However, it is important not to assume that all self-experimenters will be similarly well-informed. This is particularly so given the proliferation of ‘citizen self-experimentation’; as detailed above, we live in an era in which any sufficiently motivated citizen can engage in amateur self-experimentation using commercially available materials, informed by various sources of information on the internet. While the proliferation of scientific information has admirably broadened access to an important good for many, it has also increased the scope for misunderstanding and misinterpretation by some. There is a legitimate concern that unregulated forms of self-experimentation, perhaps by those lacking scientific or medical training, do not involve the important safeguard provided by informed consent in standard research trials.

It might also be argued that even highly knowledgeable scientific experts could fail to achieve the understanding required for an autonomous decision to self-experiment if they fail to adequately attend to the risks of their decision. For instance, it is sometimes suggested that individuals with a pressing health need who have exhausted existing treatment modalities face desperate circumstances, and that this may tempt them to take on unreasonable risks associated with an experimental treatment that they have not adequately considered.^20^ Alternatively, critics of self-experimentation might invoke concerns about undue inducement, where undue inducement is understood to arise when inducement is offered for an activity that entails more risks than a reasonable person would assume.^21^ The thought here might be that the promise of scientific glory could dazzle prospective self-experimenters in a way that prevents them from adequately considering the risks of their experiment.

We join others in maintaining a substantial degree of scepticism about these arguments outside of the context of self-experimentation.^21 22^ The fact that an individual faces desperate circumstances with limited appealing choices does not preclude the possibility that they can make autonomous choices in that context. Neither does the fact that an individual is making a decision that could substantially benefit them.^23^ Nonetheless, we will need to consider the question of whether the risks associated with self-experimentation can be reasonable. We shall return to this issue in the next section.

While a significant part of the justification for the informed consent processes is to ensure that participants have the understanding necessary to make an autonomous decision to be exposed to the risks of research, the process plausibly also plays other important roles. Bromwich and Millum point out that disclosure of information is important for preventing illegitimate control over the participant’s decision, even if the participant themselves does not understand the information.^17^ Others have claimed that the consent process signifies a relationship of trust between the participant and the research team and that the process can be important for safeguarding broader public trust in scientific research.^24^ Accordingly, even if there is little reason to doubt that a particular self-experimenter has sufficient understanding to autonomously decide to undergo an experimental intervention, there might yet be moral reasons to ensure that formal informed consent procedures are followed prior to the publication of a study.

The key question is how applicable these lines of argument are to the context of self-experimentation. We suggest that these considerations are considerably weakened when the performance and attempted publication of self-experimentation involve just one individual playing the ‘dual role’ of participant and researcher. Concerns about researchers exercising illegitimate control over a participant are surely obviated when the researcher and the participant are co-extensive. Similarly, since trust is primarily ‘a relationship between or among people’, the main purpose of which is to ‘facilitate cooperative social interactions’,^25^ it is not clear that cases of self-experimentation are best understood as involving a relationship of trust, given that one person is playing the dual role of participant and researcher. Nonetheless, these concerns arguably have more traction when the self-experimenter is part of a broader research team; in such cases, there are stronger moral reasons to ensure a robust consent process has been followed in order to safeguard against forms of illegitimate control or abuses of trust.

Moreover, we cannot foreclose the possibility that cases of published self-experimentation could affect public trust in science, whether publication is pursued individually or as part of a broader research team. However, this is a complex empirical issue, as there are many different ‘publics’ that can have quite different expectations of scientific research.^24^ Indeed, since public trust in science is significantly grounded by the scientific community’s adherence to ethical standards, the question of whether self-experimentation will undermine public trust in science is arguably secondary to the more fundamental question of whether self-experimentation is compatible with these sorts of ethical standards. As such, we shall postpone further consideration of this question until the end of the paper, where we will be able to provide a broader assessment of this more fundamental question, accommodating considerations of risk and harm to others.

### Reasonable risk

One of the reasons that it is important to distinguish between the performance question and the publication question is that there are limits to the level of risk that individuals are permitted to voluntarily expose themselves to in clinical research. Accordingly, a broadly liberal argument in favour of permitting the performance of private self-experimentation may not straight-forwardly answer the publication question.

The need for such limits on risks in research was enshrined in Article 5 of the Nuremberg Code,^26^ which states that:

No experiment should be conducted where there is an a priori reason to believe that death or disabling injury will occur…

Interestingly, though, the article also includes the following exception:

…except, perhaps, in those experiments where the experimental physicians also serve as subjects.

Here, it seems that the Code endorses the view that self-experimentation should not be subject to the same risk limits that govern standard forms of research. However, in an insightful analysis, Annas argues that the reasons for this exception can be explained by historical rather than ethical factors; in particular, the need for the prosecution in the Nuremberg trials to make a case that could not be applied to the US government, given examples of military experiments that had knowingly risked the lives of subjects—such as the Reed Commission experiments.^27^ Indeed, Annas himself rejects the exception, claiming that ‘for life-threatening research, the participation of the researcher as a subject adds nothing to the ethical analysis of whether the research can be justified at all’.^27^

This illustrates another reason to be mindful of the distinction between the performance question and the publication question. The mere performance of a ‘self-experiment’ per se may plausibly fall outside of the purview of research ethics (as long as the experimenter is not using institutional resources or assisted by others) and within the realm of self-regarding activities that might plausibly be governed by liberal ideals of non-interference. However, typical experiments are not merely ‘conducted’ as private activities—if successful, it is hoped that their results will be disseminated for the benefit of society. It is for this reason that these experiments are governed by ethical norms that seek to balance the moral value of this benefit against the interests and autonomy of research participants. Crucially, these norms are in place not just to protect participants themselves but also to safeguard trust in the scientific enterprise.^25^ When self-experimentation is presented for publication, it moves beyond the realm of private activity and the norms that govern it, and into the realm of scientific research and the norms that govern it.

It is often claimed that ethical research may only permissibly expose participants to ‘reasonable risks’. While there is considerable debate about how to define this threshold, one of us has previously outlined a conception of reasonable risk that can be adapted to the context of self-experimentation as follows (Box 1).

Consider the application of these criteria to Halassy’s case report. With respect to #1, although oncolytic viruses have a ‘tolerable safety profile’,^6^ there are some known risks to the patient of the intratumoral injection of oncolytic viruses, including bleeding and undesired metastasis at the lesion site.^28^ With respect to #2, the case report makes it clear that the viruses selected for the intervention were, in part, chosen on the basis that they have ‘documented safety in humans’. The authors point out that the measles virus used has been safely used in paediatric vaccines for decades, while VSV is described as being ‘almost non-pathogenic for humans, causing flu-like symptoms in the worst case’.^5^ Halassy’s oncologists also closely monitored the progress of the treatment and were in a position to intervene if adverse events occurred, or if the disease progressed. Accordingly, it appears that a number of steps were taken to minimise the risks of the intervention. That said, the report also highlights that the virus preparations developed for the self-experiment were research grade, rather than clinical grade—accordingly, one potential avenue for further minimising the risk of the intervention was not adopted, although the reasons for this are not clarified in the manuscript.

With respect to #3, even though it may be possible to pursue further research to better ascertain the risks of OVT, this research would plausibly have delayed the intervention; in progressive diseases such as cancer, this may jeopardise patients’ possibility of benefitting from the intervention. Finally, with respect to #4, the existing science suggested that there was at least some potential for therapeutic benefit (a potential which was happily borne out in reality). Moreover, Halassy had a life-threatening condition and few other alternative therapeutic avenues. Accordingly, it seems plausible that the potential benefit of the intervention to Halassy plausibly outweighed the risks. There was also scientific value to the study; although OVT is being investigated in robust, larger clinical studies, the case report is suggestive of the potential success of that treatment modality, and the novel approach of deploying two viruses in OVT highlights a potential avenue of research to be pursued further.

As such, it appears that a plausible case can be made that Halassy’s report of self-experimentation involved reasonable risk, on the approach outlined above. Of course, that may not be true of all self-experimentation, as the scientific and therapeutic benefits of these experimental interventions can be limited. Indeed, examples of self-experimentation typically amount to something like an *n of 1* trial. Even assuming that the experimenter follows a rigorous scientific methodology, there will often be limits to the scientific utility of the data their experiment will generate—it can be hard to generalise results from one individual, and in some cases, it can lead to misdirected conclusions.^29^ Yet, as Barry Marshall’s case shows, individual self-experimentation can still generate findings that save a huge number of lives.

### Harm to others

There is a final (overlapping) consideration that is relevant to the performance question and the publication question. Either the mere performance or the publication of self-experimentation may be ethically problematic if it exposes others to significant risk of harm.

The potential for harm to others is salient in cases in which the performance of self-experimentation involves potentially infectious agents. Notably, in Halassy’s case report, OVT can expose others to some degree of harm; since the viruses used in OVT are live, there are risks of viral shedding and of unintentional transmission to those in close proximity.^6^ However, as detailed above, the viruses deployed in the case report had a good safety profile, and it is possible to minimise risks of unintentional transmission in OVT by following protocols for the safe storage, handling and administration of oncolytic viruses.^6^ However, harms to others can also arise in a less direct sense, for example, in contexts in which self-experimentation might undermine public health measures. For instance, the RaDVaC experiment in the COVID-19 pandemic raised concerns that the self-administered homemade vaccines were less likely to be effective and yet create ‘false reassurance’ among the experimenters.^10^

Naturally, the publication of self-experimentation can also pose other kinds of risk of harm. For instance, while we might celebrate the success of OVT in Halassy’s case, one might harbour the concern that other patients could be tempted to similarly attempt unconventional therapies, perhaps before using other standard therapies.^1^ To their credit, the authors of the case report are clear about the limited generalisability of this case, and highlight that self-medication with OVT should not be the first approach to dealing with cancer.^5^ It is crucial to ensure that patients understand the limitations of what self-experimentation can teach us. Notably, though, this is an issue for the publication of *any* small-scale experimental intervention, and not just those that involve self-experimentation.

Finally, there can also be broader social concerns to consider. Zaynor’s self-experiment with CRISPR-Cas9 demonstrates that self-experimenters can now easily access ‘dual use’ technologies—technologies that have the power to significantly benefit society but that could also cause significant harm, either through misunderstanding or malice. The worry about self-experimentation using such technologies is twofold—misuse might not only cause direct harm to the individual self-experimenter, but it might also undermine societal acceptance of regulated efforts to safely develop a technology with huge therapeutic potential.

## Conclusion and an ethical decision-making algorithm

This brings us back neatly to the question of the relationship between self-experimentation and public trust in science. Ultimately, the precise nature of this relationship is an empirical issue; however, when considering the publication of self-experimentation, it is crucial to be clear about whether the self-experiment in question was performed in accordance with the ethical values and protections that the public takes to be crucial to ethical research more generally.

We believe that it is a mistake to believe that self-experimentation and the publication of its results are, in principle, necessarily ethically problematic. IRBs should remain open to the possibility that such experiments can abide by the core tenets of research ethics. However, it would also be a mistake to assume that self-experimentation by its very nature does not warrant any sort of ethical evaluation; in some cases, it may warrant the same sort of ethical oversight as conventional forms of scientific research.

When a self-experiment does not straightforwardly qualify as research, the mere performance of the activities involved can be appropriately governed by norms outside of research ethics. Nonetheless, these latter norms are crucial when we consider the ethics of publishing such studies. Accordingly, we encourage such self-experimenters to seek IRB approval where the potential publication of their experiment is foreseeable, as long as this review is timely and would not unduly jeopardise the potential benefit of the intervention.

While there are naturally significant practical challenges to regulating many forms of self-experimentation, there are good reasons to subject it to some forms of governance, including timely and receptive review by IRBs. As well as safeguarding trust, we believe that the involvement of IRBs in this way can help to ensure that the risks of the experiment to both the participant and the public are reasonable and that they are sufficiently understood by the individual experimenter. However, we have also suggested that the absence of prior IRB approval for a self-experiment does not necessarily mean that it would be unethical to publish the experiment. What ultimately matters for the publication question is whether the experiment was compatible with the norms and fundamental values that IRBs are in place to protect.

**Figure 1:**
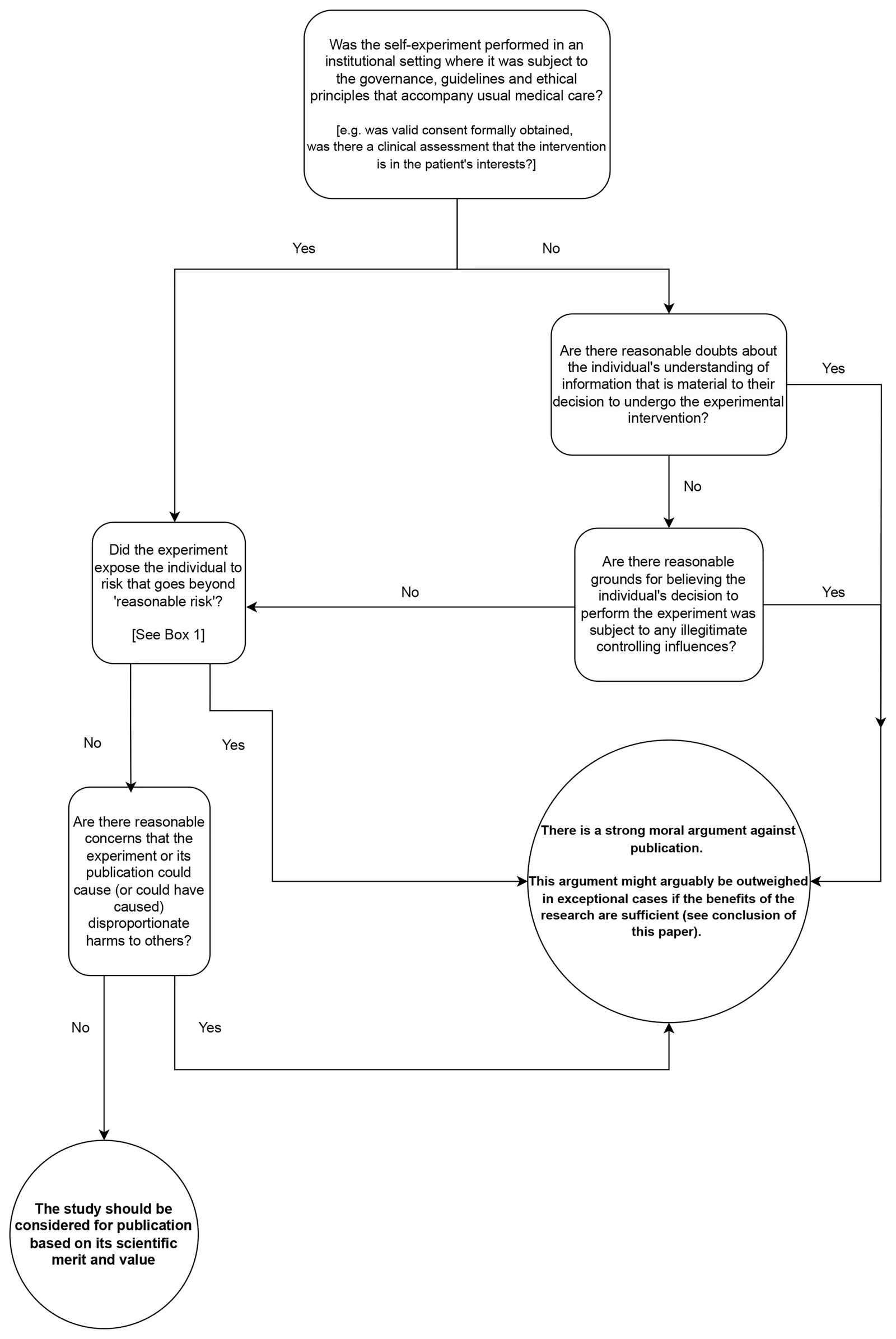
A cecision-making algorithm for publishing cases of self-experimentation

When should such studies be published? An algorithm that journal editors and reviewers might draw on to help decide in difficult cases, in which the experiment has not been subject to prior IRB review, is presented in figure 1. These are also the ethical principles that IRBs might draw on in their deliberations when confronted with a case of self-experimentation. Where a self-experiment has had IRB review that would provide supportive evidence that these ethical factors have been considered.

Finally, although our algorithm identifies that there is a strong moral reason against publishing self-experimentation when it does not meet the standards we have outlined, there may be legitimate debate about whether such research might yet be published in exceptional cases of highly valuable findings. There are broadly two approaches to this issue, reminiscent of the approach to the publication of Nazi experimental data and data from other morally abhorrent experiments.^30^ One strict deontological approach states that research that violates ethical codes should never be published.^31^ However, an alternative consequentialist or utilitarian approach may support such publication if the value of the research findings is sufficiently high. Indeed, if they were very high, there could even be a moral obligation to publish such unethical research. To take an extreme case for illustrative purposes, suppose a self-experimenter performed a highly risky experiment while suffering an acute delusional psychotic episode, undermining their decision-making capacity. Suppose that the experiment was fortunately successful and demonstrated something of considerable scientific importance. If there is sufficient value in the information gained from her self-experiment, there is still a clear consequentialist argument that might justify publication in such cases, even where there is a strong reason to think the experiment should not have been performed. We cannot hope to settle the long-standing question of precisely when (if ever) it can be ethical to publish unethical research here; however, the contribution of this paper and the accompanying algorithm is to show that this long-standing debate may not be applicable to many cases of self-experimentation.

## Data Availability

There are no data in this work.

## Box 1 Reasonable risk

In determining whether the risks of self-experimentation are reasonable, the following factors are relevant (adapted from Savulescu’s initial reasonable risk framework^32^)

1. Is there a known risk to the individual prior to commencing the experimental intervention, and what is its magnitude, based on evidence available at the time?
2. Could the risk be reduced in any other way? Is it as small as possible?
3. Could any further research be performed prior to the experimental intervention to better estimate the risk to this particular participant without jeopardising their potential to benefit from the intervention?
4. Are the potential benefits (in terms of both the participant’s health and the value of the scientific knowledge generated) of the experimental intervention worth the risks?

## Footnotes

i 1. The distinction between these sorts of questions is implicit in existing COPE advice on an anonymised case of self-experimentation, which states forum members ‘agreed that the author has the right to perform the experiments on himself, but he does not the right to have his findings published’.^12^

ii 2. There can be something of an analogue in this regard to the ethics of publishing certain kinds of case reports. For discussion, see COPE and Rosoff.^15 16^

## References

[1] Corbyn Z (2024). This scientist treated her own cancer with viruses she grew in the lab. Nature New Biol.

[2] Senior CM, Mail Online (2024). Scientist cures her stage 3 cancer with viruses she grew in the lab.

[3] Kaminska I (2021). Bioterror: the dangers of garage scientists manipulating dna. Financial Times.

[4] (2021). RaDVaC – rapid deployment vaccine collaborative.

[5] Forčić D, Mršić K, Perić-Balja M (2024). An Unconventional Case Study of Neoadjuvant Oncolytic Virotherapy for Recurrent Breast Cancer. Vaccines (Basel).

[6] Shalhout SZ, Miller DM, Emerick KS (2023). Therapy with oncolytic viruses: progress and challenges. Nat Rev Clin Oncol.

[7] Weisse AB (2012). Self-experimentation and its role in medical research. Tex Heart Inst J.

[8] Grzybowski A, Pietrzak K (2012). From patient to discoverer--Niels Ryberg Finsen (1860–1904)--the founder of phototherapy in dermatology. Clin Dermatol.

[9] Hudson Institute of Medical Research (2018). What is h.pylori and how is it linked to stomach cancer?.

[10] Guerrini CJ, Sherkow JS, Meyer MN (2020). Self-experimentation, ethics, and regulation of vaccines. Science.

[11] Smalley E (2018). FDA warns public of dangers of DIY gene therapy. Nat Biotechnol.

[12] COPE: Committee on Publication Ethics (2024). The ethics of self-experimentation.

[13] Mill JS (2003). On Liberty.

[14] Kolstoe SE, Sözüdoğru E, Messer J (2025). Is my project research? Determining which projects require review by a research ethics committee. Account Res.

[15] COPE: committee on publication ethics (2025). Ethical approval requirements for case study reports.

[16] Rosoff PM (2019). Can the Case Report Withstand Ethical Scrutiny?. Hastings Cent Rep.

[17] Bromwich D, Millum J (2013). Disclosure and Consent to Medical Research Participation. J Moral Philos.

[18] Dresser R (2019). The Reasonable Person Standard for Research Disclosure: A Reasonable Addition to the Common Rule. J Law Med Ethics.

[19] Dranseika V, Piasecki J, Waligora M (2017). Relevant Information and Informed Consent in Research: In Defense of the Subjective Standard of Disclosure. Sci Eng Ethics.

[20] Minogue BP, Palmer-Fernandez G, Udell L (1995). Individual autonomy and the double-blind controlled experiment: the case of desperate volunteers. J Med Philos.

[21] Emanuel EJ (2005). Undue inducement: nonsense on stilts?. Am J Bioeth.

[22] Allmark P, Mason S (2006). Should desperate volunteers be included in randomised controlled trials?. J Med Ethics.

[23] Autonomy PJ (2020). Rationality, and contemporary bioethics.

[24] Resnik DB (2018). The ethics of research with human subjects: protecting people, advancing science, promoting trust.

[25] Resnik DB (2011). Scientific research and the public trust. Sci Eng Ethics.

[26] (1996). The Nuremberg Code (1947). BMJ.

[27] Annas GJ (2010). Self experimentation and the Nuremberg Code. BMJ.

[28] Lin D, Shen Y, Liang T (2023). Oncolytic virotherapy: basic principles, recent advances and future directions. Signal Transduct Target Ther.

[29] Lakhtakia R, Burney I (2015). Self-Experimenting Physicians. Sultan Qaboos Univ Med J.

[30] Higgins WC, Rogers WA, Ballantyne A (2020). Against the use and publication of contemporary unethical research: the case of Chinese transplant research. J Med Ethics.

[31] Ridley A (1995). Ill-gotten gains: on the use of results from unethical experiments in medicine. Public Aff Q.

[32] Savulescu J (1998). Commentary: safety of participants in non-therapeutic research must be ensured. BMJ.

